# An overview and bibliometric analysis on the colorectal cancer therapy by magnetic functionalized nanoparticles for the responsive and targeted drug delivery

**DOI:** 10.1186/s12951-021-01150-6

**Published:** 2021-11-29

**Authors:** Mahdieh Darroudi, Mehrdad Gholami, Majid Rezayi, Majid Khazaei

**Affiliations:** 1 Department of Medical Biotechnology and Nanotechnology, School of Science, Mashhad University of Medical Science, Mashhad, Iran; 2 Department of Physiology, Faculty of Medicine, Mashhad University of Medical Science, Mashhad, Iran; 3 Department of Chemistry, Marvdasht Branch, Islamic Azad University, P.O. Box 465, Marvdasht, Iran; 4 Medical Toxicology Research Center, Mashhad University of Medical Science, Mashhad, Iran; 5 Metabolic Syndrome Research Center, Mashhad University of Medical Science, Mashhad, Iran

**Keywords:** Drug-delivery, Anticancer drugs, Magnetic nanoparticles, Bibliometric analysis, Colon cancer

## Abstract

With the growing demands for personalized medicine and medical devices, nanomedicine is a modern scientific field, and research continues to apply nanomaterials for therapeutic and damaged tissue diagnosis. In this regard, substantial progress has been made in synthesizing magnetic nanoparticles with desired sizes, chemical composition, morphologies, and surface chemistry. Among these materials, nanomagnetic iron oxides have demonstrated promise as unique drug delivery carriers due to cancer treatment. This carrier could lead to responsive properties to a specific trigger, including heat, pH, alternative magnetic field, or even enzymes, through functionalization and coating of magnetic nanoparticles, along with biocompatibility, good chemical stability, easy functionalization, simple processing, and ability to localize to the tumor site with the assistance of external magnetic field. Current studies have focused on magnetic nanoparticles’ utilities in cancer therapy, especially for colorectal cancer. Additionally, a bibliometric investigation was performed on the public trends in the field of the magnetic nanoparticle to drug delivery and anticancer, which represented progressing applications of these carriers in the multidisciplinary zones with a general view on future research and identified potential opportunities and challenges. Furthermore, we outline the current challenges and forthcoming research perspective for high performance and fostering advanced MNPs in colorectal cancer treatment.

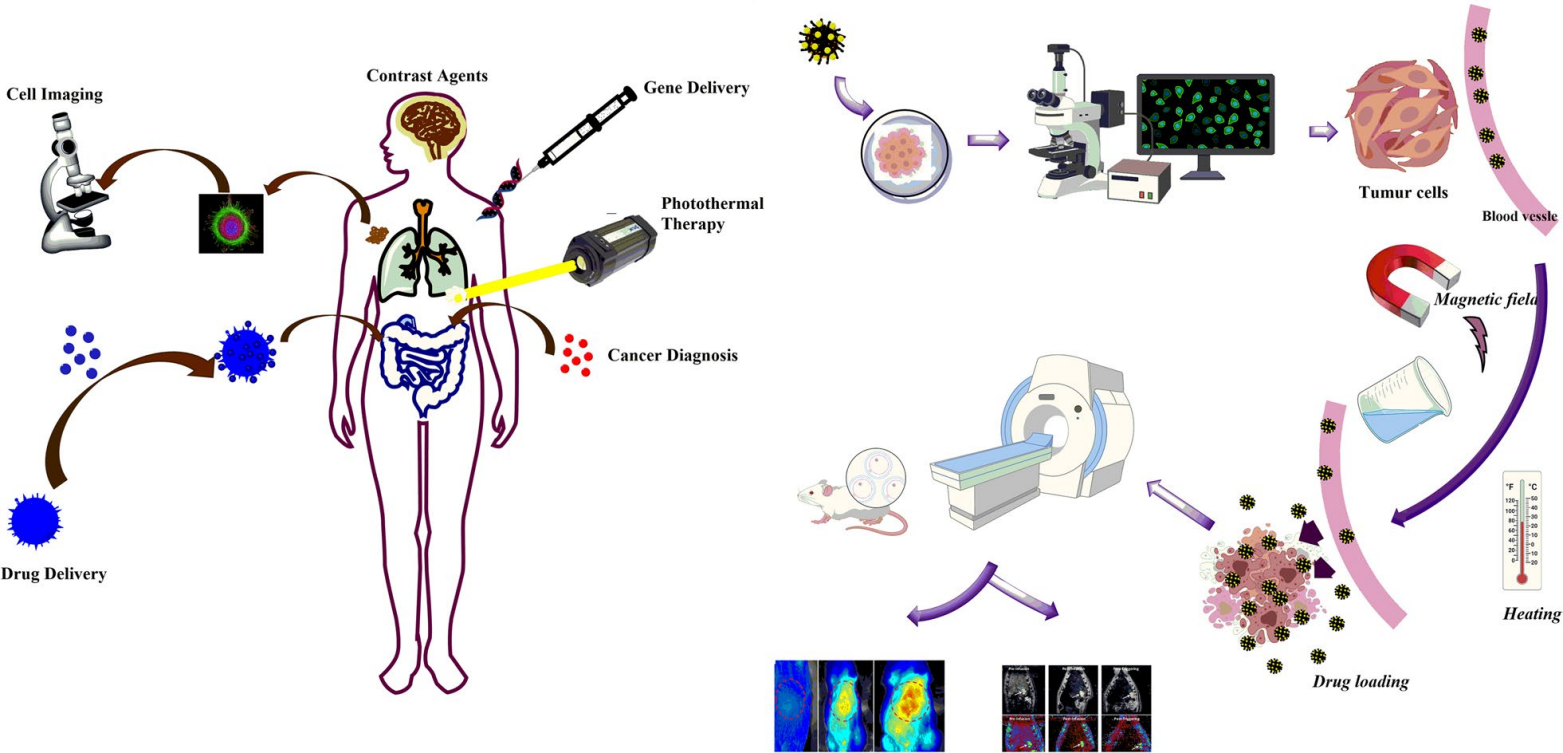

## Background

The development of an effective disease treatment has been a significant goal of the human for more than a millennium. Our knowledge of the body and the function of different cooperating components in the human body has improved our knowledge about living cells. This information and understanding would lead to developing a wide range of medications, systematic disorders, inflammatory illnesses, and body malfunction’ components. The need for a novel and more effective treatment is predominantly robust throughout disorders or the appearance of a new disease that poses a worldwide threat with a very tangible risk as we have faced these days for causing a pandemic or when drugs become ineffective due to the resistance mechanism evolution [1].

**Fig. 1:**
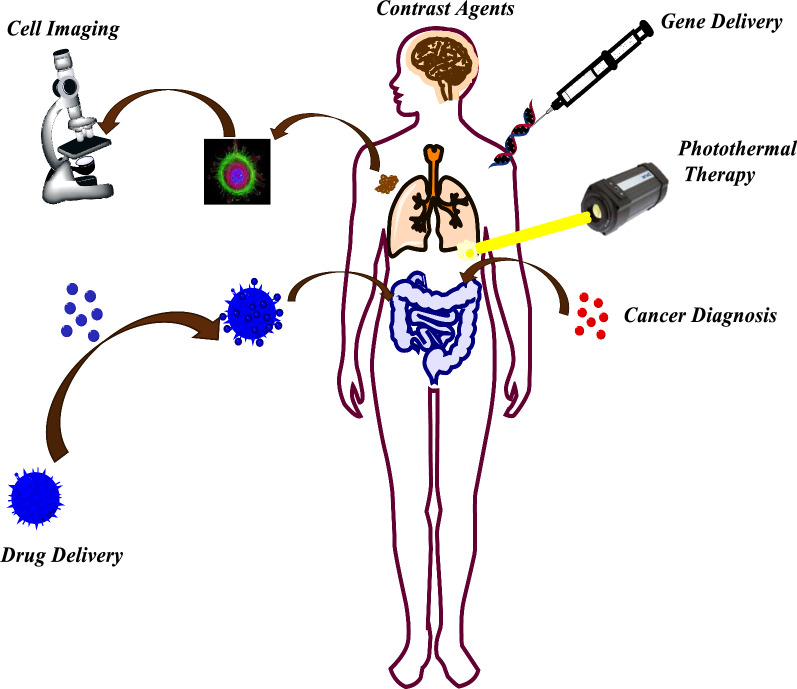
Use of Magnetic nanoparticles in medicine

Cancer is known as one of the most dangerous and devastating diseases posing a severe threat to millions of individuals' health and lives with rising worldwide incidence and mortality rates [2]. The “cancer” term refers to the uncontrolled growth of cells and multiplication owing to a cell phenotype producing growth signals and unresponsive to anti-growth signals. These kinds of cells have evaded apoptosis, unrestricted replicative potential, induced angiogenesis, and stimulated invasion and metastasis [3]. There are various cancer types with few common or typical features; therefore, its treatment is precisely challenging. Researchers worldwide have an urgent demand to develop safe, novel, and efficient agents for treating various types of cancers [4]. Recently, numerous treatment strategies, including surgery, chemotherapy [5], radiation therapy [6], targeted therapy [7], immunotherapy [8], and hormonal therapy [8], have been employed to fight against cancer (Fig. 1) [9]. The efficacy of conventional chemotherapy has been decreased by the non-specific distribution and rapid clearance of anticancer drugs, low efficiency, drug resistance at the cellular and tumor level, and every so often considerable toxicity of common anticancer agents when prescribed at higher doses. Subsequently, the enormous effort has been devoted to considering the cellular and molecular mechanism of these diseases and the design of drugs for their treatment, which has driven the exploration of innovative nanomedicine that could overwhelm the foremost downsides of common cancer treatments by specific attacking properties and mechanism.

Various types of carriers for drugs have been established, including nanoparticles, dendrimers, polymeric micelles, viral nanoparticles, liposomes, carbon-based systems, silica, magnetic nanoparticles, and gold nanoparticles [1]. Nanomaterials have subjected the whole world in the modern era surrounding domestic resources to electronic and medical sectors [10, 11]. The development of nanomaterial chemistry has produced nanoparticle carriers with controllable physicochemical properties and narrow size distributions that could be exploited for various purposes, such as enhancing the treatment's efficiency or monitoring its effects. With advances in molecular and cellular biology, these signs of progress suggest chances produce sophisticated selectivity targeted-nanomedicines based on carriers and carriers conjugated with numerous biologically active compounds such as nucleotides, drugs, enzymes, genes, or proteins. Such materials could potentially form the basis of very specific, safe, and efficient cancer treatments [12]. Recently, nanomaterials’ utilizations received a very substantial role in the drug delivery system [13]. The drug delivery through nanoparticles has led researchers worldwide to focus on developing life-saving nanoparticles [14]. Magnetic nanoparticles (MNPs) based on metals such as iron [15], nickel [16, 17], or metal oxides [18] have aided modern technology's efficient development [19]. MNPs can find various biomedical applications, including simultaneously therapy and diagnosis [20]; hence, the enormous interest in MNPs efficiency can be easily understood [21]. On the other hand, MNPs could be used broadly as drug carrier devices from their capability of raising the water solubility of hydrophobic drugs suppress or eliminate fast renal excursion (biocompatibility), good chemical stability, easy functionalization, simple processing [22, 23], enhancement in an organ or cell-specific drug accumulation with the aid of external magnetic field where the therapeutic effect is needed in tumor cells [24, 25].

This review aims to comprehensively cover recent advances in the field of pure or modified MNPs with noncovalently or covalently attached drugs for colorectal cancer treatment. Generally, we have classified the uses of MNPs into three primary concepts. In “Quantitative approaches” section, a general bibliometric analysis is employed to evaluate the outcome of using MNPs in drug delivery for colorectal cancer and the significance of MNPs in CRC treatment during the last 40 years. “Main text” section deals with using MNPs with covalent polymer-drug conjugates in both in vitro and in vivo studies in detail.

## Quantitative approaches

Cancer is one of the most significant death causes after heart and infectious diseases, despite some cancer treatment breakthroughs [26]. Current drawbacks of conventional cancer therapies are biological barrier clearance [27], poor bio-distribution [28], and non-specific drug delivery mode of action [29], limiting overall effectiveness [30–34]. Nanotechnology can overcome these obstacles through engineered nanomedicine as nanoparticle conjugated drugs [35]. Among many probable usages of nanoparticles lies the area of drug delivery; they can deliver large quantities of drugs or other medical cargoes due to the large surface area of nanoparticles [36, 37], then the emergence of beneficial drug delivery systems is one of the vital factors [38]. Furthermore, the nano agents for theranostics of cancer have emerged as a promising research area that could integrate the treatment and diagnosis of cancer by combining nanoplatforms with therapeutic agents to enhance tumor-specific targeted drug accumulation in cancer cells, leaving the normal cells unaltered [39]. The main concern is improving the efficiency of drug delivery procedures, generally defined as sustainability, low disruptions, and precise and accurate targeted delivery control [40]. Recently, drug delivery systems based on magnetic nanoparticles have been investigated, and several have been accomplished in this field. Regarding the recent studies in drug delivery systems, many methods have been proposed [41, 42].

This section focused on the publication trends of research in CRC treatment based on MNPs using a bibliometric approach. Bibliometrics refers to implementing statistic methods for evaluating research productivity for countries, institutes, and individuals as well as measuring academic performance [43]. However, bibliometric is insufficient in assessing a research area outputs, and it should include other ranges of inputs such as a literature review to discover the insight of publications trends [44, 45]. This research explores this field of study's research status by bibliometric and qualitative literature reviews from the past to the current year. According to the publication trend identified, research in using MNPs in systemic drug delivery for CRC treatment is a hotspot; consequently, this section would offer an overview of the current status and future directions in this field.

### The strategy of search

**Fig. 2:**
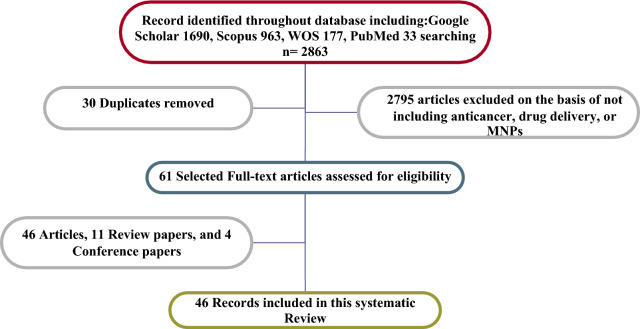
Prisma flow diagram

**Fig. 3:**
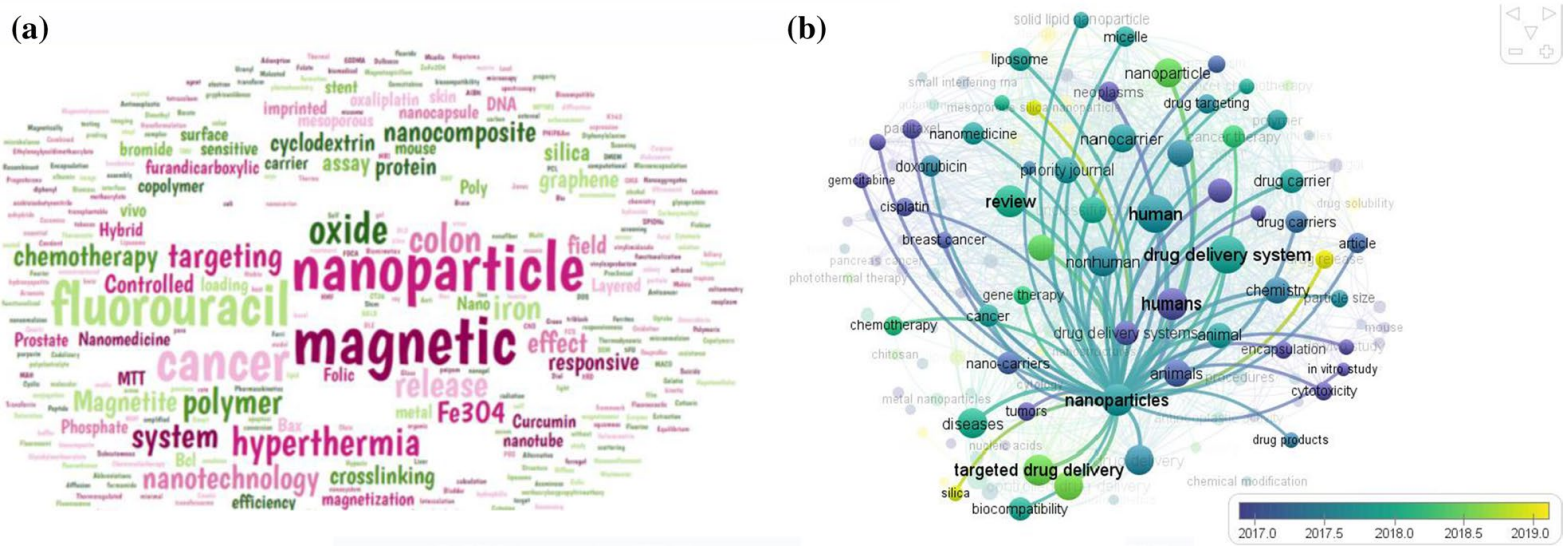
**a** The network of keywords and **b** clustering of keywords during the time period

The bibliometric search terms were performed in Google Scholar, Scopus, PubMed, and Web of Science Core Collections (n = 2863) from 1965 to 2021. The data was obtained through the pre-analysis of retrieval results from the online version of the core collection in Web of Science on 7th February 2021 [46]. The words “Drug delivery” AND “Anticancer” identify all articles related to anticancer drugs in the retrieval time range from 1990 to 2021 that contain the keyword in the title list, and 3007 publications were encountered. After excluding literature types to “Colon cancer,” a total of 177 articles were detected, which was distributed in the period of 2002–2021. Since the emergence of relevant literature in 1991, the number of literature has rocketed, and the average annual number of published articles has exceeded since 2012. Also, articles using the keyword “Magnetic nanoparticle” OR “MNP” OR “Magnetic nanocarrier” to identify all articles contain the keyword in the title which, 36,407 publications met the selection criteria. Upon further screening, only 30 publications were categorized through “Drug delivery” AND “Colon cancer*” OR “Anticancer” AND “Magnetic nanoparticles” keywords that were utilized for further analysis, which the search returned 27 articles from the WOS database (Fig. 2). Also, the Google Scholar and PubMed databases were carried out in bibliometric studies, in which search terms resulted in 1690 and 33 publications, respectively. The keyword search terms in SCOPUS were used to retrieve the data. After careful inspection, a total of 61 publications were identified as suitable for subsequent analysis from the SCOPUS database. The search returned 46 articles, 11 review papers, and 4 conference papers. Besides, Index Keywords capture an article’s content with greater depth and variety [43]. In this study, there are 412 Keywords. Figure 3 illustrates the keywords plus named “Magnetic nanoparticles”, “Delivery”, “Cancer”, “Fluorouracil”, and “Drug” have been used by many top authors of this work who are experts on colon cancer treatment. It can be found from Fig. 3a that “Magnetic nanoparticle” and “Anticancer” have been attractive keywords in this area. In contrast, there are a few attempts to use “oxaliplatin” in their articles. Moreover, Fig. 3a displays the correlation between 100 Index Keywords. This graph is drawn by the VOSviewer package, a tool for comprehensive science mapping analysis for quantitative research in bibliometrics [47, 48]. The network of institutions includes 54 clusters, 203 nodes, and 189 links (Fig. 3b).

### Annual publications

**Fig. 4:**
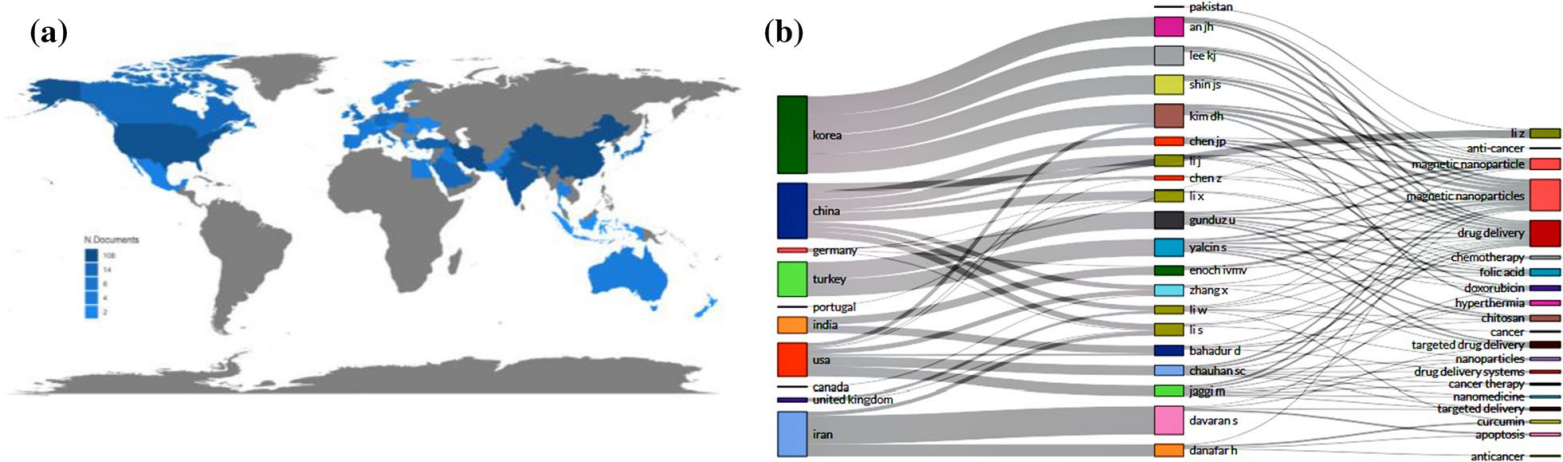
**a** Country collaboration map-related documents-published in the considered period, **b** three-field plot of top-author, top-countries; and top authors keywords on the considered topic

Figure 4a gives a complete picture of magnetic nanoparticles’ usages all around the world. This repartition-guided such important data for scientists to discover where they should work and build up some cooperation. Figure 4b exhibited that between 20 countries Korea, China, Iran, and United States have the most significant number of published articles compared to other nations from the data in the graph. Korea has specialized studies earlier than other countries with the most significant number of normalized strong collaboration links to other countries with the rate of 20% of publication. China, Iran, and United States have 15%, 13%, and 13%, respectively, after Korea in the mentioned topic.

### Geographical analysis on contributing countries and institutions

Bibliometrics provides another proper function to plot a Sankey diagram to visualize multiple attributes at the same time. A Three-field plot (Sankey Diagram) listing the respective authors, Keywords, and country on the considered topic is shown in Fig. 4b. This figure shows the relationship among top countries, top authors, and top author's keywords. The top five countries in which these documents were published included “Korea” (9 DOC), “China” (7 Docs), “Iran” (6 Docs), “Turkey” (6 Doc), and “The United States” (6 Docs), and the top five authors are Davaran S. Kim DH, Shin JS, Gunduz U, and Lee KJ. Articles are classified into different subject categories in the SCUPOS database. Figure 4a, b, visually demonstrate the co-occurring subject categories network with six nodes and 167 links.

### Most productive and influential authors

**Fig. 5:**
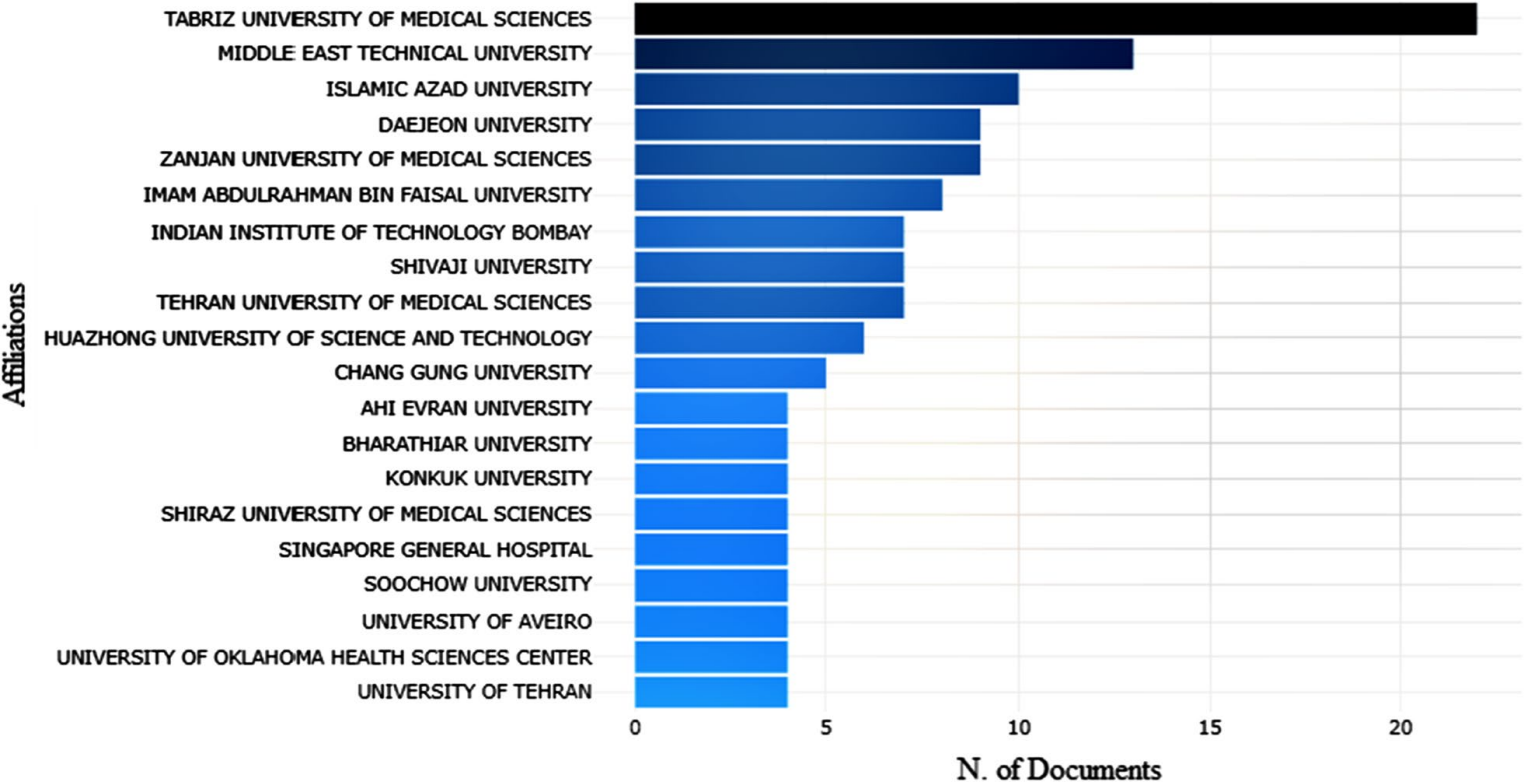
The most relevant affiliations on the considered topic

The countries’ network was generated to analyze the distribution of articles on anticancer drugs for colon cancer research in magnetic nanoparticles. Figure 5 indicates the co-authorship network, which was pruned from R Studio. Concerning the collaborative relationships, some research communities have been identified. Tabriz University of Medical science, Middle East University and Daejeon University published the most articles.

**Fig. 6:**
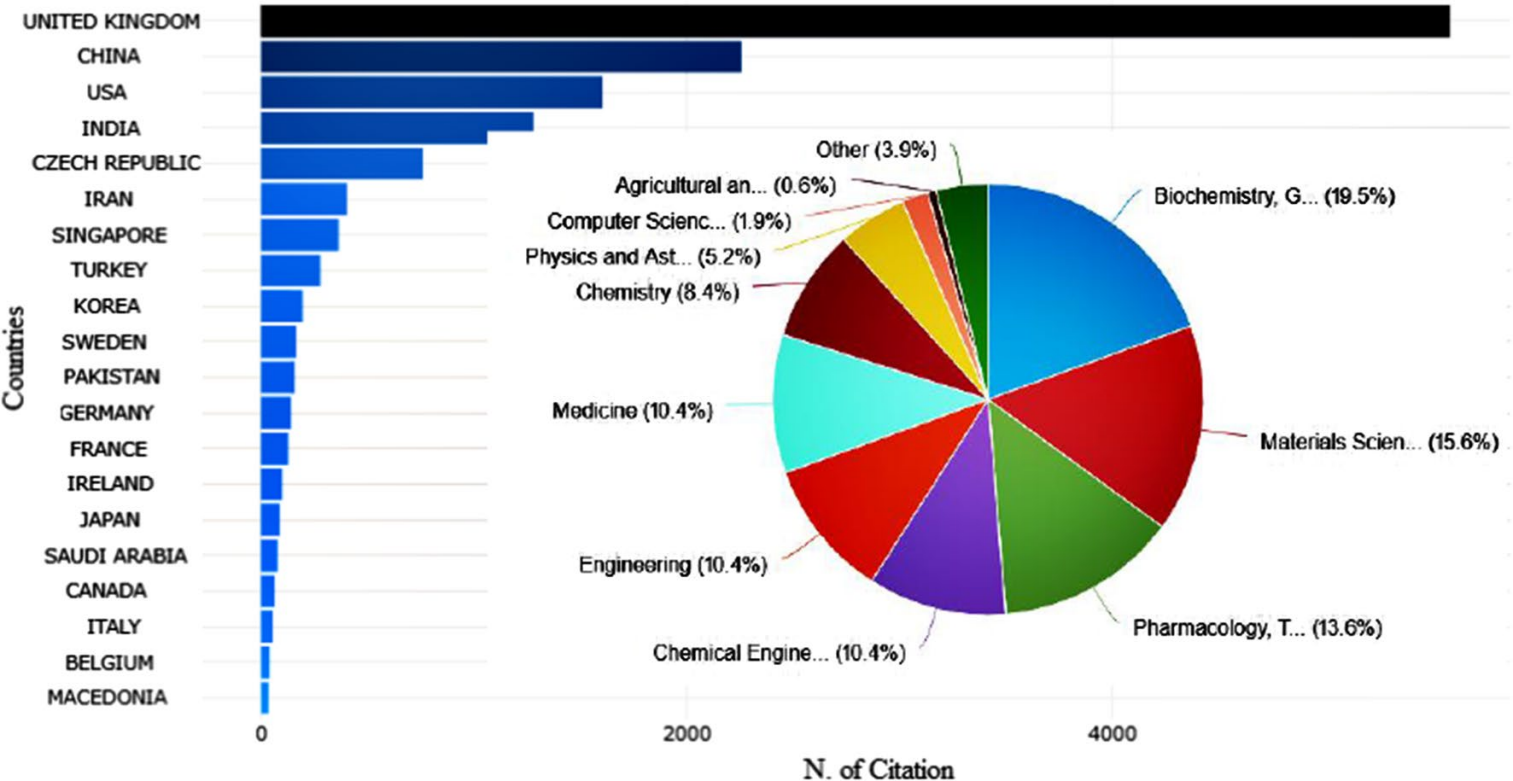
The most cited countries on considered topic, inset: the bibliometric analysis by subject area

Furthermore, Fig. 6 displays the trend of bibliometric analysis by the most cited countries in this area. The most cited countries’ trend demonstrated that the research theme was very active in United Kingdom, China, United States, Indian, and Iran. They can be regarded as research centers of colon cancer research based on magnetic nanoparticles, indicating much attention among scholars due to the enhancement of different types of cancer. The majority of publication on colon cancer treatment based on magnetic nanoparticles is done in Biochemistry, Genetics and Molecular biology, Material science, Pharmacology, Toxicology, and Pharmaceutics, which have the most extensive studies on this carrier by 19.5%, 15.6%, and 13.6%.

### Research hotspot of 2021 and beyond

**Fig. 7:**
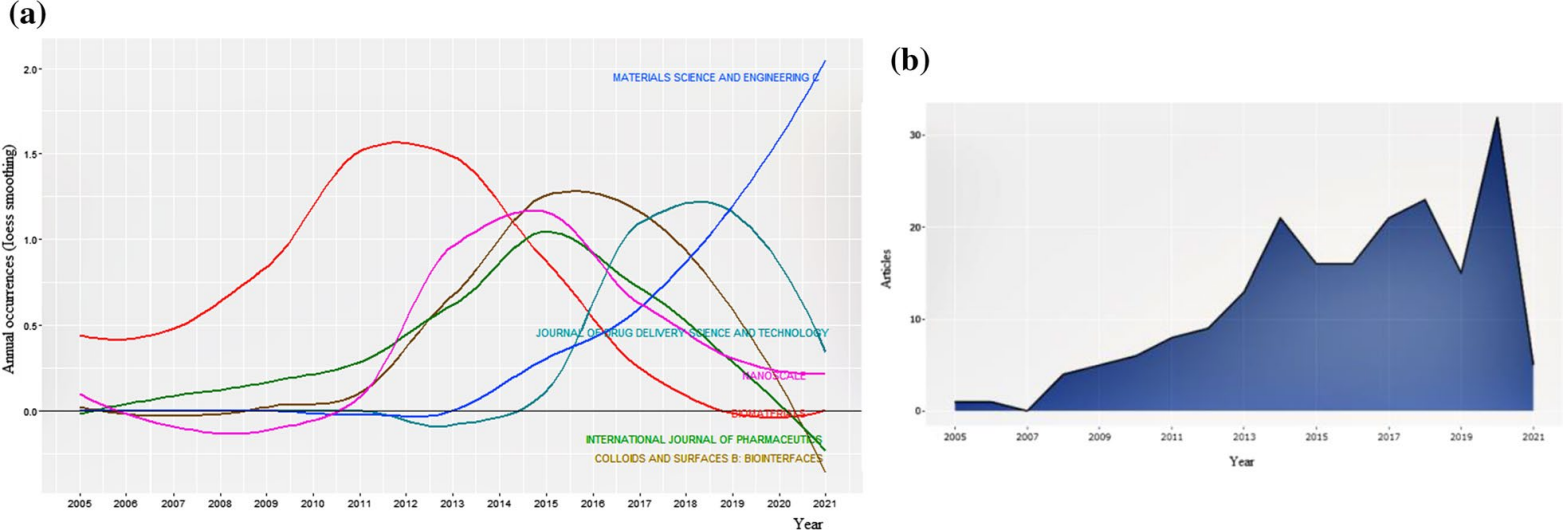
**a** The plot of world growth of considered topic based on the subject, and **b** annual scientific publications sorted by publication years

Moreover, the evaluation of the considered topic based on the subject area is plotted in Fig. 7a. The majority of publications on the considered topic are found frequently in articles and refer to the process used to make documents findable in databases. This figure exhibits that top subjects’ index growth is varied during the considered time. We see that Material Science and Engineering has the most extensive study on this topic since 2006.

Figure 7b demonstrates the dispensation of published articles between 2005 and 2021, in which the trend of publications grows gradually with an upward curve from one published article in 2005 to about 10 articles in 2013. There was a rocket around 2013, reaching approximately 14 publications in that year. It is shown an unexpected decrease in 2015 coming down, but afterward should be better in 2016, reducing the number of publications has been compensated. The number of published articles has reached nearly 30 in 2020, 45% of all articles published at all times.

## Main text

Despite some significant breakthroughs in cancer treatment, cancer is one of the most pivotal causes of death in patients after infectious and heart disease [49, 50]. Colon cancer is one of the most common kinds of cancer globally, which usually developed from focal changes in the colon epithelial tissue to a cancerous polyp with genetic alterations and their accumulation in cancer development [51, 52]. Surgical resection of the primary tumor is the main treatment for colorectal cancer, followed by adjuvant chemotherapy [53]. Targeted therapy could deliver the therapeutic agents directly to the colon. Then, the drug concentration would be increased in the colonial tissue, resulting in dose reduction; this therapy mainly involves using monoclonal angiogenesis inhibitors and antibodies to circumvent the tumor cells. The most significant pros of targeted drug delivery over chemotherapy would be reducing side effects due to its specificity. The FDA has approved 16 drugs for the colorectal cancer treatment, out of which 12 drugs [5-fluorouracil (5-FU), Oxaliplatin, irinotecan, leucovorin, regorafenil, capecitabine, trifluridine, tipiracil, cetuximab, aflibercept, panitumumab, and bevacizumb] have been administered as main drugs in chemotherapy. Currently, setbacks have observed with conventional chemotherapeutic methods including poor solubility, reduced permeability, low accumulation into cancerous cell, non-specific targeting, and dose-dependent toxicity on the normal tissues [54]. In the case of colorectal cancer, the physiology of the colon additionally poses a hurdle for conventional oral dosing of anticancer drugs, owing to active pharmaceutical ingredients being protected from the gastric environment of the stomach and intestines, making it challenging in drug delivery. On the other hand, drug delivery technology has witnessed significant developments over the last few years [5, 55]. The nano-drug delivery system has emerged as a prospective therapy and diagnostic tool to address the conventional chemotherapy agents’ problems [56]. The drug delivery concept entails transferring a specific dose of various therapeutic agents as natural and synthetic drugs, proteins, and genes to the desired binding site within a predetermined time using various devices or specific formulas [57, 58]. As well, drug carriers protect the medicinal agent, enhance the pharmaceutical effect, and carry lipophilic and hydrophilic drugs to meet the system’s anticipated usage. Targeted drug delivery is considered as an approach to delivering therapeutic agents to an intended tissue or organ to reduce toxicity and increase efficacy [42, 59]. Nanotechnology can overcome this obstacle through engineered nanomedicine as nanoparticle conjugated drugs [60].

Various nanomaterials have been fabricated for cancer treatment and diagnosis, permitting the drug to bypass the immune system. It has been exhibited that the relationship between nanomaterials and innate immune system responses depends on various parameters, including shape, size, surface modification [61]. The concept of magnetic drug delivery applies an external magnetic field to drive a drug carrier with a magnetic property to an intended position in the body. Among different magnetic nanoparticles (MNPs) classes, magnetic iron oxide is one of the most used magnetic materials, especially in biomedical applications [28]. MNP is considered to be one of the promising materials for biomedical applications. Coating them with biocompatible and biodegradable materials is crucial to reducing their potential toxicity and protecting their magnetic core from corrosion. Moreover, biopolymers can release the absorbed drugs at a rate determined by their degradation [62].

**Fig. 8:**
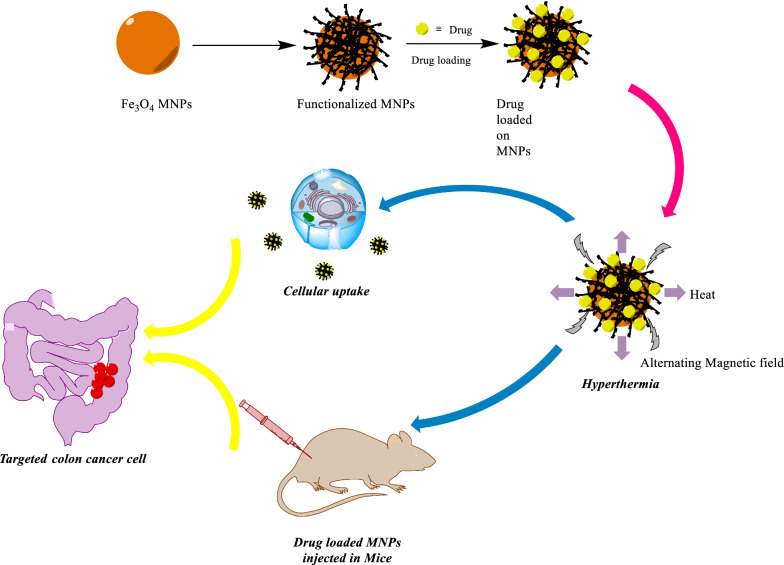
Drug loading process on the magnetic NPs, stimuli sensitive release of drugs upon applying external stimuli and schematic illustration description the in vivo and in vitro nano combination therapy

**Fig. 9:**
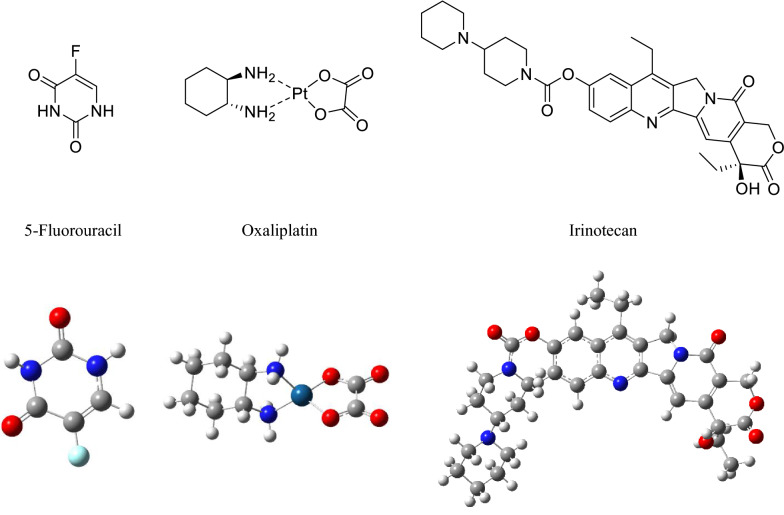
Structure of 3 most common anticancer drugs 5-fluorouracil (5-FU), oxaliplatin, and irinotecan for colon cancer by magnetic nanoparticles

With the advent of MNPs, targeting NPs for site-specific delivery has become an achievable task. Magnetic NPs fulfill several prerequisites such as controllable drug release, adequate high magnetic moments, chemical stability, and low toxicity in a physiological environment as well (Fig. 8) [63, 64]. In the near future, researchs may develop a specific, targeted magnetic nanocarrier containing a cocktail of drugs or vaccines showing efficacy in targeted treating colorectal cancer. Nowadays, the most used colorectal chemotherapeutic drugs are 5-fluorouracil (5-FU), Oxaliplatin, irinotecan, and Capecitabine which are examined for targeted drug delivery based on magnetic nanocarrier (Fig. 9) [65]. These drugs could also be used in combination, mainly due to their synergistic effects and better prognosis [66].

### Magnetic core–shell nanocarrier

**Fig. 10:**
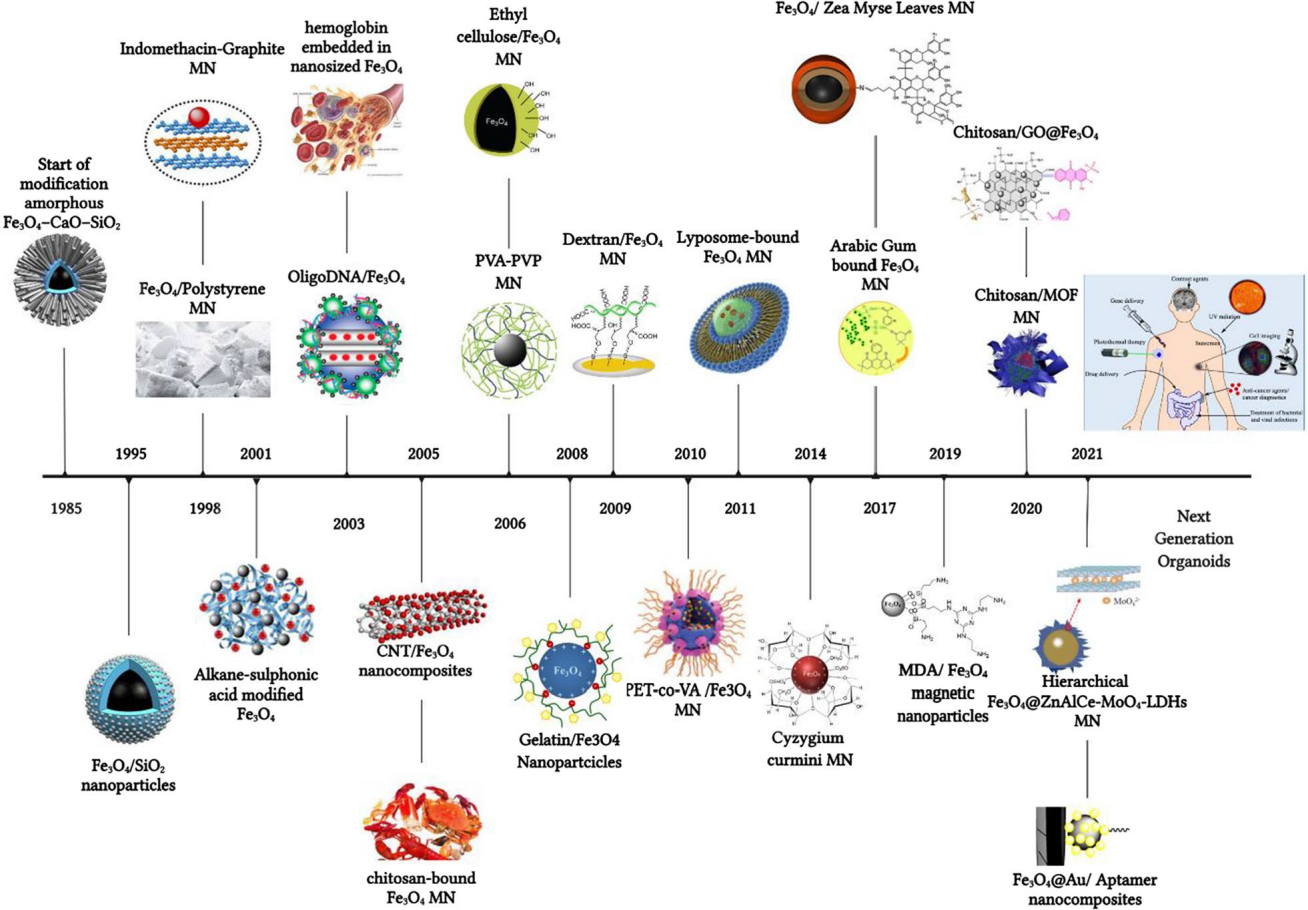
The timeline of magnetic nanoparticles in therapeutic and imaging application

Magnetite Fe_3_O_4_ NPs are an interesting class of materials due to their ease of separation and relatively nontoxic nature. The modification and functionalization of NPs with different kinds of macromolecules, polymers, biomolecules, etc. have been subjected to different attempts during the last decades, as shown in Fig. 10 [67–82]. Significantly, the development of such environmental and benign green nanocarrier diminishes the cost of chemical transformation and furnishes ecological welfares appropriate for immobilizing drugs and enzymes. For considering an efficient drug carrier, the material should be contained in unoccupied sites to carry the therapeutic target and drug loading enhancement [7]. Most research has recently focused on evolving efficient drug delivery resources that could improve the cell internalization of drugs and lessen cytotoxicity. Furthermore, a magnetic drug nanocarrier should have distinct properties, including low toxicity, magnetization, and appropriate drug release and uptake [83–85]. Despite available synthesis techniques of MNPs, the intended MNPs could have low magnetization, mainly when targeting around deep tissues in the body. Therefore, a new approach must be integrated to reach better results [86]. It was demanded that using AMF arose in deeper penetration and localized in cancer cells, and it can be beneficial in triggering the releasing of drugs from encapsulated MNPs [87]. Hence, it addresses various magnetic drug nanocarriers to encapsulate drugs, which will be discussed in the following sections.

### Targetting magnetic drug delivery

Generally, hyperthermia is not considered a unique application for MNPs in cancer treatment, especially colorectal cancer. Much attention has been gained to the increasing usage of these NPs in drug delivery systems, which allow better control for the release of drug upon reaching the cancerous site and for guiding the nanocarrier due to external triggering of the magnetic field. Furthermore, these strategies could lead to incorporate single anticancer chemotherapeutic drugs up to multichemotherapeutic drugs. Because of a broad range of possibilities for combining various functions in cancer treatment, great attention has been attracted to utilizing MNPs for application in cancer therapy, especially here discussed on colorectal cancer. These methods sometimes bring the combination of promising properties in cancer treatment, including photothermal, drug target, photodynamic therapy, hyperthermia, pH-responsive carrier, and more permeability and retention in the drug delivery process, discussing in the following sections.

### 5-Fluorouracil (5-FU)

**Fig. 11:**
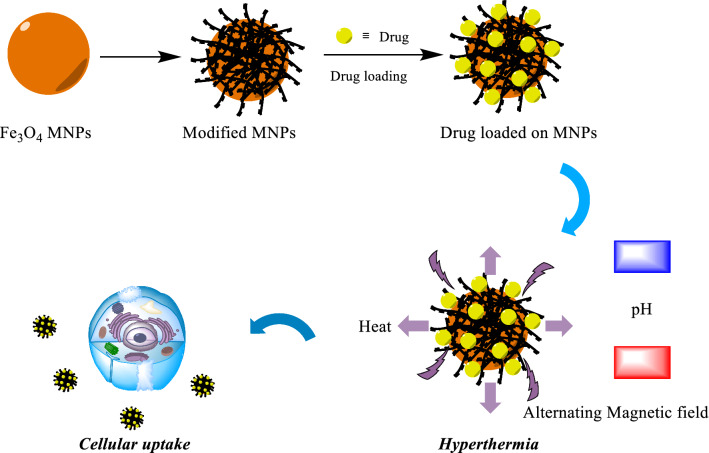
Drug loading and sensitive release of drugs upon applying external stimuli

One of the most used anticancer drugs and clinical radiosensitizer is 5-FU. 5-FU is a fluorinated pyrimidine analog with demonstrated antitumor activity against adenocarcinomas arising in the gastrointestinal tract and breast cancer and ovary and against squamous cell carcinomas arising in the cell to 5-fluorodexyuridine monophosphate [88]. It can covalently bind to thymidylate synthase in the presence of methylenehydrofolate, inhibition of the enzyme loading to depletion of deoxythymidine triphosphate, and interference with DNA synthesis and repair [89]. 5-FU could be metabolized to 5-fluorouridine triphosphate, which is incorporated into RNA. One or both of these metabolites has been shown to account for the cytotoxicity of 5-FU in experimental models [90]. The sensitivity of drug loading and drug-releasing process upon the usage of external stimuli used in drug delivery of anticancer drugs is displayed in Fig. 11.

Magneto liposome NPs loaded with 5-FU were developed by a reproducible thin film hydration technique [91]. It was suggested that superparamagnetic iron oxide NPs could be embedded into a multilamellar lipid vehicle. No cytotoxicity in human colon fibroblast CCD-18 and human colon carcinoma T-84 cell lines and acceptable hemocompatibility of these core–shell NPs were proved. They established an excellent drug loading value and sustainable release profile, which 5-FU would trigger by the healing properties and hyperthermia of NPs on drug release. This magneto liposome structure could confer responsiveness to magnetic gradients, giving both the possibility of selective drug-delivery and adequate heating characteristics for hyperthermia effect. As a result, nanoformulation could constitute a potential candidate for combined antitumor therapy against colon cancer. An adequate amount of 5-FU could be selectively accumulated into the tumor mass accompanied by a hyperthermia effect. Moreover, the incorporation of 5-FU was studied by set-up into the nanoparticulate matrix and surface deposition onto already formed magneto liposome particles.

A new nanocomposite would be used as a carrier for 5-FU was using the folic acid (FA) as a modifier on the Fe_3_O_4_@chitosan surface found manifold at the cancer cells' surface in comparison with normal cells [92]. Through loading 5-FU on the surface of Fe_3_O_4_@chitosan-FA MNPs, the circulation time, controlled release of drug and adequately specify for target size, and suitability of MNP system for drug delivery were investigated. This nanocarrier was used toward bladder carcinoma T24 cells as a cancer cell line. Apoptosis and cell viability were measured, indicating proposed MNPs have a remarkable drug loading efficiency and had an excellent antitumor activity without any adverse outcome for human bladder epithelial cell HBlEpC as normal cell lines. The apoptosis induction and cytotoxicity effect studies on both cancer lees T24 and normal cells HBlEpC displayed a specific effect of 5-FU loaded on MNPs. One of the dominant parts of this work was the selectivity behavior of 5-FU drug release and the impressive killing of the cancer cells. Also, it had remarkable biocompatibility with a negligible effect on cell proliferation decreasing.

A magnetoliposome NPs loaded with 5-FU, a superparamagnetic magnetite nucleus embedded into a multilamellar lipid vesicle, and the drug release behavior of 5-FU was studied to define heating properties [93]. Also, cytotoxicity in the absence of human colon carcinoma, T-84 cell lines, and colon fibroblast CCD-18, and hemocompatibility of these MNPs were studied. It was reported that these 5-FU MNPs released drugs by hyperthermia-triggered drug release. The cytotoxicity of MNPs was studied in human colon fibroblast CCD-18 and human colon carcinoma T-84 cell lines during 5 days. The magnetoliposome MNPs tested hyperthermia and magnetically targeted delivery for a combined antitumor therapy against colon cancer exhibited a dominant release mechanism.

The usage of Fe_3_O_4_@polyetheleneglycol-(Mg/Al) L.D.H. MNPs, and Fe_3_O_4_@polyetheleneglycol-(Zn/Al) L.D.H. MNPs were examined as a carrier for 5-FU, and it was demonstrated that cytotoxicity of proposed MNPs was more than free 5-FU against liver cancer HepG2 cells along with less toxic for normal fibroblast 3T3 cells [94]. These MNPs could use as the multifunctional carrier to target drug delivery by an external magnetic field and exploited as hyperthermia for cancer cells HepG2 and the chemotherapy efficacy.

A new temperature-sensitive hybrid microgel Fe_3_O_4_@poly(isopropyl acrylamide) MNPs using acrylic acid, butanoic acid, or allylamine as well as crosslinking with a magnetic response examined for controlled release of 5-FU and Oxaliplatin (OXA) [95]. To determine the drug encapsulation efficiency, drug delivery-release and cytotoxicity were examined against T-84 colon cancer cells. Drug release performed against PBS, obtaining a good activity above the LCST, exhibited high biocompatibility and good capacity to load drug against colon cancer cells besides high sensitivity to the temperature alteration. It was demonstrated that 5-FU + pNIPAM@Fe_3_O_4_-Butanoicacid reduced 57% relative inhibition of proliferation on the T-84 colon cancer cells growth pNIPAM-co-arylamine@Fe_3_O_4_-acrylic acid MNPs in the presence of external magnetic field produces a slight migration of HCT15 cells in suspension. The temperature sensitivity consented to enhance drug release and enhance cytotoxic therapy in the colon tumor cells.

Apoptosis induction in human colon adenocarcinoma cell lines HT-29 and HCT-116 were studied by ionizing radiation and 5-FU-loaded on the triblock copolymer PEG-PBA-PEG MNPs [96]. The 5-FU-loaded on the magnetic triblock copolymer Fe_3_O_4_@PEG-PBA-PEG MNPs, investigating in combination treatment with 5-FU, and also was examined that 5-FU encapsulated MNPs, which had a stronger effect on the enhancement of therapeutic efficacy of CRC treatment. It was caused by the induction of killing cell Bcl-2, which apoptotic and necrotic cell damage through treatment process proposed to be due to radiosensitivity of MNPs, and more accumulation of the chemotherapeutic drug in the cells and also the cumulative effect of 5-FU and radiation. It was expressed that the proposed MNPs had a strong effect on Bcl-2 decreasing and Baz increasing, cleaved caspase-9, cleaved caspase-3, cleaved PARP in comparison with each treatment lonely. The usage of the magnetic triblock copolymer as a carrier for 5-FU and irradiation work by triggering apoptosis improves in vitro treatment efficacy and is expected as a practical approach for colon cancer treatment.

A recent study on drug delivery of 5-FU was recently performed to find out usage stimuli sensitive system through 5-Fu encapsulated in copolymer Fe_3_O_4_@pectin-*g*-poly(dimethyl aminoethyl methacrylate) MNPs as a multi sensitive thermal, pH, and magnetic field carrier [97]. It was examined the drug release of 5-FU in vitro using L929 fibroblast cells and MCF-7 cancer cells. It was reported that by altering the pH (7.4 and 5.5), the temperature (37 and 44 °C) and the presence of the magnetic field exhibited sensitivity toward pH. More drug release exhibited (56% vs. 40%), and at pH 7.4 exhibited more drug release (63% than 45%), and in the presence of the magnetic field, the 5-FU release was increased to 100%.

Mohammadi et al. used iron oxide nanoparticles (Fe_3_O_4_) nanoparticles to enhance the drug delivery into the cells, and also, they examined the effect of 5-FU loaded in the PLGA coated Fe_3_O_4_ NPs and radiation on the DNA damage levels in the monolayer culture of DU145 prostate carcinoma cell line [98]. They were exhibited that DU145 cells were cultured and treated with different concentrations of 5-FU and NPs as 5-FU carriers. After treatment, the induced damages of DNA were examined by alkaline comet assay. They displayed that DNA damaged raised as long as the increase of the free 5-FU concentration and PLGA coated Fe_3_O_4_ NPs concentrations as a carrier of 5-FU in combination with X-ray. The extend of DNA damage induction from 5-FU in polymeric coated Fe_3_O_4_ NPs combined with 2 Gy of megavoltage X-ray was more than free 5-FU. It was reported that the drug-loaded NPs could deliver 5-FU more efficiently into the cell, and MNPs could be stable and effective drug-delivery vehicles for 5-FU.

Khoei et al*.* reported an investigation on the uptake and cytotoxic effect of polycaprolactone/chitosan/nanographene oxide coated on Fe_3_O_4_ MNPs as a carrier for 5-FU, and then studied radiofrequency hyperthermia, using the alternate magnetic field on proliferation capacity of CT26 colon cancer cell [99]. The dialysis bag method was used to measure the 5-FU drug release from functionalized MNPs, and it resulted in enhanced cellular uptake with elevated MNPs concentrations. It was exhibited that the proliferation capacity of cells diminished in combination with radiofrequency hyperthermia and the extent of reduction in colony number. The prepared MNP carrier efficiently delivered 5-FU into CT26 cells.

An examination of the effectiveness of a combined therapeutic approach using MNPs to induce magnetic hyperthermia and 5-FU based chemotherapy was performed and named thermochemotherapy [100]. The thermo-chemotherapeutic approach containing the intratumoral application of functionalized Fe_3_O_4_@Chitosan using 5-FU and magnetic hyperthermia prospectively enhances the human colon cancer HT29 treatment. The utility of the human colon cancer cells HT29 heterotopic tumor model in mice exhibited that thermo-chemotherapeutic treatment was more effective in inactivating colorectal cancer than tumor treatment alone. It also impacted tumor volume and tumor cell proliferation compared with single therapy modalities and affected DNA replication and reparation as measured by H2AX and phosphorylated H2AX expression. Moreover, the 5-FU-Fe_3_O_4_@Chitosan did not distinctly induce apoptosis nor necroptosis of target cells, caspases non-conspicuous phosphorylated-RIP3, besides that it could be rendered tumor cells surviving therapy sensitively in rather than other therapeutical methods. The designed system enabled the relapse-free eradication of colorectal cancer. It demonstrated lessening proliferation potential, broad damaged DNA in the treated tumor cells, and reduced tumor volume, inducing by local combinatorial thermo-chemotherapy in vivo.

Several studies have been performed to develop sustained drug-delivery of 5-FU using various NPs; another study was performed to synthesize 5-FU loaded PLGA magnetic nanocapsule to investigate nanocarrier’s potent to deliver therapeutic agent for tumor-targeted therapies through dialysis method in vivo and in vitro. It was established that the entrapment of anticancer drugs in nanocapsules could control the release profile of medicines. Shakeri-Zadeh et al. [101] proposed a method for preparing a 5-FU loaded magnetic PLGA nanocapsule and explored the potential of 5-FU loaded magnetic NPs as the carrier in cancer chemotherapy agents in vivo. This 5-FU loaded magnetic nanocapsule demonstrated a longer lifetime in the plasma of rabbits compared with free 5-FU and excellent antitumour activity against the colon cancer allografts and reduced the adverse side effects of 5-FU therapy improving the therapeutic index.

Engineered bacterial cells could be implemented to prompt therapeutic enzymes, and also induction of recombinant enzyme expression could be controlled by external triggers [102]. The immunogenicity could overcome cells in immunoprotective matrices that could support cell function and survival by encapsulation. They reported triggering enzyme-prodrug therapy with MNPs through isolative microencapsulation of Escherichia coli strain overexpressing the cytosine deaminase enzyme upon thermal induction transcriptional control of thermoregulatory λpL-cI857 promoter providing thermal switch in order to triggered enzyme synthesis. The engineered cell was co-encapsulated with Fe_3_O_4_ MNP.s in alginate microcapsule; consequently, cytosine deaminase expression could remotely trigger activation by alternating magnetic field-induced hyperthermia. It was proposed that the enzymatic conversion of 5-fluorocytosine (5-FC) to 5-fluorouracil (5-FU) and subsequent killing of cancer cells line could be initiated by remote activation of Fe_3_O_4_ MNPs that co-encapsulated with the bacterial expression host. This enzyme-prodrug therapy would ultimately yield an improved therapeutic index relative to monotherapy, as alternating magnetic field mediated hyperthermia could be anticipated to pre-sensitize tumors to chemotherapy under proper conditions.

**Fig. 12:**
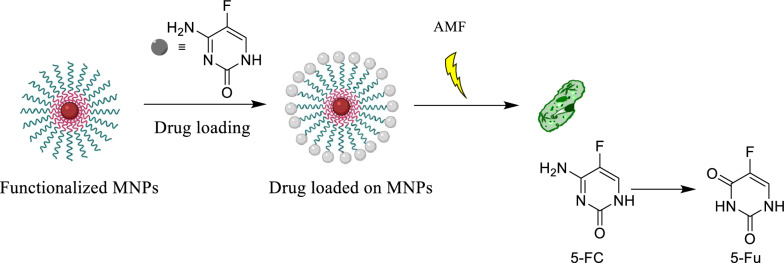
The schematic of prodrug 5-FC in drug delivery based on magnetic nanocarrier

Rice LB proposed that stem cell-based gene therapy combined the migratory activation of stem cells through tumor cell cytokine with genetic modification to deliver specialized cytotoxic activities to the tumor [103]. Tumour-targeting of cytosine deaminase expressing stem cells combined with 5-FC administration showed therapeutic activity in the colon’s carcinoma both in vitro and in vivo, which shows that the cytosine deaminase combined with 5-FC had an acceptably safe. They also studied the retroviral transduction to breast cancer with bacterial cytosine deaminase, resulting in significant sensitivity to the cytotoxic effect of 5-FC in vitro (Fig. 12). The tumor-targeted delivery and a site-specific production of 5-FU by cytosine deaminase expressing cells can apply a strong cytotoxic effect on rapidly proliferating tumor cells. MNP core coated in an aqueous solution exhibited significantly good r2 relaxivity and also acceptable r2/r1 ratio and suggested that modified MNPs could be a good T2/T1 agent. Moreover, immunohistochemistry and molecular imaging could confirm the migration of F3-cytosine deaminase cells toward tumor cells and displayed the cytotoxic action as cellular vehicles for cancer chemotherapy murine model for prostate cancer. The systemically controlled cells have gotten the tumor formation site and reduced tumor size in the presence of 5-FC.

A comparison between polylactic-co-glycolic acid (PLGA)-Fe_3_O_4_ MNPs carrier for 5-FU and without carrier was performed in the spheroid model on the proliferation and viability of the human colon cancer cell line HT-29 [104]. The different concentration of 5-FU loaded on nanoparticles and 5-FU was studied on HT-29 spheroid cells, and hyperthermia was performed. The effect of treatments was evaluated using colony formation assay for proliferation capacity and trypan blue dye exclusion test for cell viability. It was reported that during hyperthermia, 5-FU loaded on MNPs has more enhanced the cytotoxic effect than the controlled group with 5-FU and 5-FU loaded on PLGA NPs, and could raise the intracellular concentration of drug in the cancer cell. Combined with 5-FU loaded on Fe_3_O_4_ MNPs encapsulated with PLGA, it could absorb thermal energy, therefore, locally increasing the temperature inside tumor cells and remarkably reducing the proliferation capacity of HT-29 cells. Therefore, the temperature in heated cells is higher than those that do not contain MNPs, resulting in promoting cell death by hyperthermia and enhancing the drug release. They demonstrated that the MNPs core could act as a thermosensitizer and increase the hyperthermia cytotoxic effects. It was enhanced the usefulness of treatments and diminish the 5-FU dose needed for tumor control.

### Oxaliplatin

The Pt-based drug oxaliplatin forms an adduct with genomic DNA that can ultimately lead to cell apoptosis; after intravenous injection, the Pt drug is excreted with a fraction remaining, limiting the amount of drug that can only bind to DNA combination of chemotherapy and radiotherapy is an effective therapeutic method, displaying Oxaliplatin sensitize the cell to radiation.

Initially, Oxaliplatin encapsulated in Fe_3_O_4_ and pectin crosslinked with Ca^2+^ (MP-OHP) was used for potential targeted drug delivery in colorectal cancer by Kumar Dutta et al. [105]. The in vitro drug release was considered at different pH, and also, its application as targeted drug delivery was assayed against cancer cells. The drug encapsulation efficiency was proposed to be 55.2%, and the drug loading amount was determined to be 0.10 wt% in MP-OHP nanocarrier; and also, a sustainable release of Oxaliplatin was exhibited at pH 5.5 and 7.4, where the drug release profile fulfilled a combination of swelling and diffusion-controlled mechanism. The cytotoxicity effect of magnetic nanocarrier tested on MIA-PaCa-2 cancer cell line, and also it displayed 10-folds higher cytotoxicity than the equivalent concentration of the free drug.

In another attempt, biomimetic MNPs mediated by MamC encapsulated Oxaliplatin was used for chemotherapy against colon cancer [106]. It was supposed that electrostatic interactions between Oxaliplatin and biomimetic MNPs triggered the nano assembly’s formation and stabilized the physiological pH. At acidic pH values, the biomimetic MNPs became neutral, and the Oxaliplatin was released, and it is suggested as a great potential by hyperthermia. Also, it was proposed that the fast internalization of nano assembly by tumor cells by endocytosis led to enhanced toxicity to higher levels compared with the soluble drug. Moreover, the biomimetic MNPs could be cytocompatible and non-hemolytic, providing positive colon cancer effects and a promising nano assembly for targeted chemotherapy against this tumor.

Beatriz Garcia-Pinel performed one of the first try-outs for the local delivery of Oxaliplatin, which proposed a magnetic nanocarrier biomimetic magnetoliposome for Oxaliplatin studied its application on colon cancer. It was exhibited to efficiently reduce the IC_50_ in comparison with that of free Oxaliplatin [93]. The strong interaction with macrophages displayed toxicity and aggregation possibility leading to viability decrease of the cell. While these MamC-mediated biomimetic MNPs coupled with Oxaliplatin enveloped in a more pegylated liposome, it could improve the couple’s biocompatibility and cellular uptake Oxa-BMNPs without tangible decrease cytotoxic activity in colon cancer cells. It was reported that hemolysis lessened from 5 to 2%, which could be hemocompatible. The agglutination of red blood cells reduced, along with the elimination of toxicity in white blood cells.

Recently, a new study on drug delivery of Oxaliplatin in colon cancer treatment was performed through CD-44 targeting receptor for HCT-116 cancer cell lines [107]. The Fe_3_O_4_/mesoporous silica-NH_2_ MNPs functionalized by amine groups were used as a magnetic nanocarrier for Oxaliplatin. The value of drug loading-release was measured at different levels pH 5, and 7.4 respectively, for cancer cells and human blood conditions. The MTT assay and IC_50_ displayed a decrease for the proposed pH-responsive Fe_3_O_4_/mesoporous silica-NH_2_/oxaliplatin compared to free Oxaliplatin. It was observed since CD-44 mediated endocytosis by amine group bonding resulting in higher intracellular uptake and CD44-binding and enhancing cytotoxicity.

**Fig. 13:**
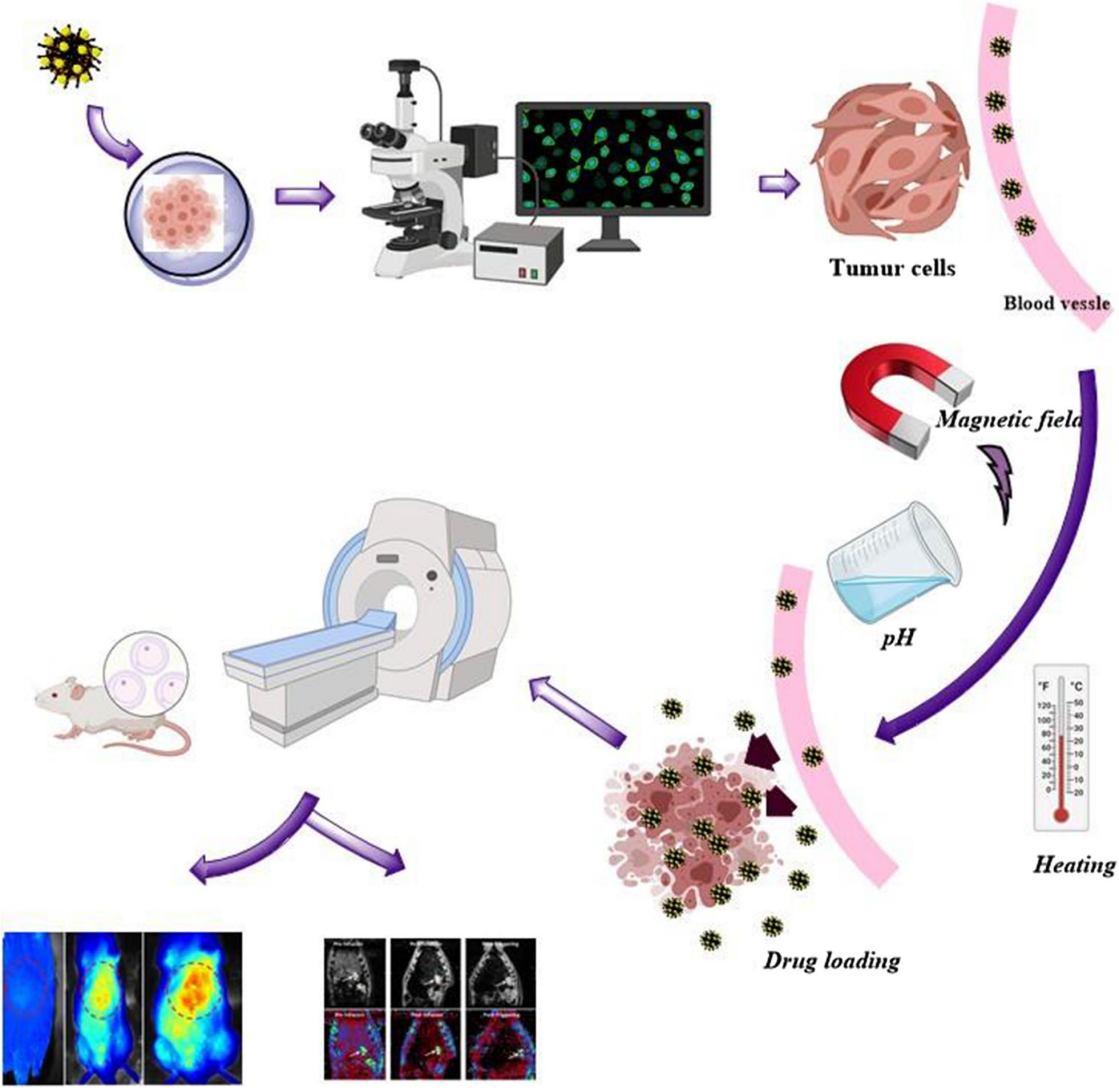
The schematic representation of tumor treatment through stimuli sensitive release of drugs by magnetic nanocarrier and in vivo and in vitro

The first attempt in vivo utilizing MNPs as a carrier for Oxaliplatin was performed to improve the colon cancer treatment responses by Liu et al. [108]. It was demonstrated that Au-Fe_3_O_4_-Herceptin MNPs as a carrier for Oxaliplatin acted as dual-functional NPs conjugated and could oxaliplatin delivery and human epithelial growth factor receptor 2 targetings (HER2). The prepared oxaliplatin-Au-Fe_3_O_4_-Herceptin was proposed as a promising multifunctional stand for concurrent HER2 targeted chemotherapy and magnetic traceable for colon cancer. Drug release studies were performed by dialysis cassettes and exhibited that 25% of the drug could release at pH = 8, while more than 58% released at pH = 6 in 4 h incubation, which indicated the pH-dependent release of designed magnetic nanocarrier of Oxaliplatin. The active targeting specific delivery of oxaliplatin-Au-Fe_3_O_4_-Herceptin MNPs to HER2 in vivo study was performed in a subcutaneous xenograft mouse model contained SGC-7901 cells through detecting aggregated low intensity in T_2_-weighted magnetic resonance images, and also further confirmed by immunohistochemistry. It was supposed that employing a designed platform; it could raise the efficacy and lessen the side effect of oxaliplatin chemotherapy (Fig. 13).

Localization and triggering the release of Oxaliplatin to colorectal cancer treatment was performed on the hybrid liposome-MNPs formulation loaded with Cy5.5 dye as the carrier for Oxaliplatin and CC-531 cell viability [109]. Then rats were orthotopically implanted with CC-531 cell lines treated with liposome-MNPs encapsulated Oxaliplatin, treated with the alternating magnetic field. Drug delivery was assessed by optical and magnetic resonance imaging. Biodistribution was performed to determine the oxaliplatin delivery. The result showed that the hybrid liposome-MNPs nanocarrier significantly increased the oxaliplatin release by almost 18%, along with lower cell viability after alternating magnetic field (< 0.001). Magnetic resonance imaging on mesenteric vein injected animals exhibited R2 changes in the tumor regions after infusion compared to the surrounding cells. A large tumor necrotic zone and enhancement in the survival rates were noted in the mesenteric vein injected animals. The alternating magnetic field triggered site-selective drug delivery at a high concentration and enhanced survival outcomes in colorectal cancer-bearing rats.

### Irinotecan

Irinotecan is also one of the most common cytotoxic chemotherapy drugs used to treat metastatic colorectal cancer since it has a good potential and a relatively preferable antitumor activity. Several clinical studies have focused on treating colorectal cancer based on irinotecan on the magnetic carriers [110].

In this regard, tumor hypoxia became a more predominant problem for diagnosis as well as a cancer treatment due to difficulties in drug delivery by chemotherapy to the cancer regions with a reduced vasculature and oxygen supplies [111]. External physical stimulus mediated drug delivery magnetic field could be effective in targeted drug delivery. The treatment of colon cancer in the presence of irinotecan delivery by MNPs was studied, and the effect of simultaneous exposure of low-intensity ultrasound and static magnetic field on hepatocellular (HepG2) and colon cancer (HCT116) carcinoma cell inhibition was assessed in vitro. It was reported that the treatment with MNPs carrier significantly increased cell inhibition up to 86% in comparison with 50% inhibition with the free drug, and appeared more efficacy on HepG2 cells rather than HCT116 cells during the initial 24 h, and reduced cancer cell necrosis without any inhibitory effect on healthy cells (MC3T3). It was suggested that it had strong application potential for cancer treatment in the lower dosage of drugs to achieve similar inhibition to reduce health risks accompanied by drugs.

One of the first attempts to deliver irinotecan-based magnetic nanocarrier was performed by Serra et al. by introducing mesoporous magnetic core–shell Au functionalized with thiol-poly (ethylene glycol) and loading of irinotecan [112]. It was reported that the magnetic nanorod exhibited a high drug-loading ability and also magnetic behavior allowing the controlled drug release, with high cellular viability of HeLa cells. By applying an alternating magnetic field due to both the effect of mechanical damage of cell by MN rods and the effect of irinotecan release from the nanocarrier affected by a magnetic field, which dramatically reduces the amount of both drug and magnetic nanorods needed to efficacy destroy cancer cells.

To have an effective drug delivery system, recently, a recent attempt on targeted drug delivery based on chitosan-based polyelectrolyte complexes with the orientation of Fe_3_O_4_, enabling the targeting delivery of the first-line model drug for irinotecan to the tumor region under a magnetic field [113]. The designed MNPs displayed high drug encapsulation capacity and enhanced anti-colon cancer cell efficacy compared to the bare drug. The magnetic nano chitosan-based polyelectrolyte complexes showed effective internalization through cancer cells and favorable targeting ability via in vivo. It was proposed that magnetically targeted drug delivery chitosan-based polyelectrolyte complexes MNPs systems could afford a promising platform to overwhelmed conventional chemotherapy's side-effects for colorectal cancer.

### Capecitabine

In general, capecitabine is another anticancer drug that is known as a hydrophilic prodrug with the ability to be converted to the 5-FU in tissues of the body by enzymatic processes to improve the intratumor drug concentration and the tolerability through tumor-specific conversion to the active drug [114]. Also, it is largely prescribed for a patient who is involved in metastatic breast cancer and colorectal cancer. Ghadiri et al. investigated the encapsulation of capecitabine and MNPs in dextran-spermine NPs for drug delivery to the cancerous cells. The aim is that the synthesized encapsulated MNPs would have more cytotoxicity than free capecitabine through transporting NPs to U87MG glioblastoma cells with fewer side effects [115]. One of the first attempts on the capecitabine was encapsulation by ionic gelation to formulate a drug delivery carrier to recognize the cancerous cells with more efficacy, then studied the physicochemical properties and in vitro cellular uptake and release profile. It was exhibited that at the optimized condition with 26.1%, the efficacy of encapsulation drug release at neutral pH would be 56%, and at acidic pH would reach 98%. The cytotoxicity assessment showed that encapsulated capecitabine on the MNPs would be more toxic than free drugs. Significant cellular uptake was demonstrated by U87MG glioblastoma cells using Prussian blue staining and TEM. It was suggested that the prepared magnetic carrier would be suitable for encapsulation and delivery of hydrophilic drugs in the treatment of cancerous cells. Also, since this carrier had a positive charge, the uptake of drugs by cancerous cells would be improved.

In brief, this review focused on colorectal cancer therapy, emphasizing magnetic nanoparticles due to technological benefits and manipulation by the magnetic field. Magnetic Nanoparticles have been extensively utilized in target drug delivery, hyperthermia, extraction of biomolecules, imaging, and a significant tool for cancer treatment. The morphology of MNPs has drawn tremendous attention from different scientific fields and researchers because of their properties such as biocompatibility, nontoxicity, unique surface chemistry, and particular inducible magnetic moment. Since colorectal cancer is a leading cause of death among cancers worldwide, our review featured recent studies accomplishments made in colorectal cancer treatment by means of MNPs. The first part of our review gave a bibliometric overview of MNPs in CRC treatment and a comprehensive review of using these carriers in treating CRC and drug targeting. The final part was included the drugs using MNPs as a carrier loading different anticancer drugs for CRC treatment. Furthermore, the conclusion section outlines the current challenges and future research perspective for high performance and fostering advanced MNPs in colorectal cancer treatment.

## Conclusion

In recent years, the use of MNPs as a controlled drug delivery system has received much more attention. Moreover, various therapeutic procedures based on magnetic nanocarriers for CRC treatment are currently under development, which was included in MNPs carriers and its sophisticated stimuli-responsive classification in both in vitro and in vivo studies. It was verified that the publication trend of research in systemic drug delivery systems is being a hotspot. The increasing incidence of colon cancer over the last years and its inadequate response to chemotherapy establish the main reasons for the interest being placed on the biodegradable magnetic nanocarrier for the delivery system. The main advantages of these magnetic nanoformulations are stimuli-responsive, highly controllable drug release based on the external magnetic field, high solubility, bioavailability, and stability. They could be administered through several routes that could improve treatment by hyperthermia, pH, or thermal sensitivity. An alternating magnetic field shows promising results in colon cancer therapy. The potential to combine tumor targeting and controllable drug delivery gain great scope for designing therapies with desirable pharmacodynamic and pharmacokinetic properties representing a crucial step toward personalized medicine. The practical application of the drug delivery system in CRC could be facilitated through an enhanced focus on the processes and requirements of therapeutic development and preclinical evaluation, including regulatory requirements. However, concerning magnetically guided nanoparticles, an efficient in-vivo drug delivery system is still elusive, with vital problems being drop off in the strong magnetic field with distance inside the body and ever slighter. Promising in vivo studies’ results have also been reported for preclinical trials in the case of cancer therapy.

Overall, some critical issues should be addressed before the translation to clinical applications. The most critical issue could be the gap between the lab production of these magnetic carriers and their large-scale synthetic production, mainly in nanoparticles. The drug delivery system with the acceptable potential for clinical translation has not to be very complex due to the high rate of failing possibilities during upscaling of the preparation process. Furthermore, the drug-carrier conjugates should balance the pharmacokinetic advantages. Another vital challenge could be the degradation, controllable drug-release and extraction of drug delivery pathway from the body, toxicity studies, and much more data from in vitro, in vivo, to clinical trials are still required to introduce the best targeting system to the commercial medical practice.

## Data Availability

All the data sharing not applicable to this article as no dataset was generated or analyzed during the current study.
